# Primary Cutaneous CD4⁺ Small/Medium T-cell Lymphoproliferative Disorder With T-cell Receptor (TCR) Clonality: The Importance of Clinical Context in Management

**DOI:** 10.7759/cureus.108913

**Published:** 2026-05-15

**Authors:** Sri Naidnur, Coral Martes-Villalobos, Sonia A Neave, Emily DeSantis, Rick Lin

**Affiliations:** 1 Dermatology, Oasis Dermatology Group, McAllen, USA; 2 Dermatology, Boston University Chobanian and Avedisian School of Medicine, Boston, USA; 3 Dermatology, Universidad Central del Caribe School of Medicine, Bayamon, PRI; 4 Dermatology, HCA Healthcare Corpus Christi Medical Center Bay Area, McAllen, USA; 5 Dermatopathology, Sagis Diagnostics, Houston, USA

**Keywords:** cd3, cd4, clinicopathologic correlation, cutaneous t-cell lymphoma, dermatopathology, gene rearrangement clonality analysis, immunohistochemistry (ihc), pd-1, primary cutaneous cd4+ small/medium t-cell lymphoproliferative disorder (pcsm-lpd), pseudolymphoma

## Abstract

Primary cutaneous CD4+ small/medium T-cell lymphoproliferative disorder (PCSM-TCLPD) is an uncommon, indolent lymphoid proliferation that can resemble more aggressive cutaneous T-cell lymphomas. Recognizing this entity is important to avoid unnecessary workup and treatment. We report a case of PCSM-TCLPD with T-cell receptor (TCR) clonality in a 59-year-old Hispanic female who presented with a six-month history of a slowly enlarging nodule on the left forehead, associated with intermittent pruritus and mild tenderness without bleeding or ulceration. She denied systemic symptoms, including fevers, night sweats, or weight loss. Histopathologic and immunophenotypic findings supported the diagnosis in the appropriate clinical setting. Laboratory studies, imaging, and bone marrow analysis demonstrated no evidence of systemic involvement. Given the lesion’s location in a cosmetically sensitive area, care was coordinated with dermatology, hematology/oncology, and plastic surgery. This case highlights the importance of clinicopathologic correlation in distinguishing PCSM-TCLPD from its malignant mimickers and in guiding appropriate management.

## Introduction

Primary cutaneous CD4+ small/medium T-cell lymphoproliferative disorder (PCSM-TCLPD) is an indolent cutaneous T-cell lymphoproliferative disorder that was reclassified in 2018 from lymphoma to lymphoproliferative disorder because of its typically benign clinical behavior and uncertain malignant potential [1,2]. It most often presents as a solitary papule or nodule on the head and neck, particularly the face, with less frequent involvement of the trunk and extremities [1-3]. Although most cases occur in adults, pediatric presentations have been reported but remain uncommon [3,4].

Despite its favorable prognosis, PCSM-TCLPD can be diagnostically challenging because it overlaps clinically and histopathologically with reactive lymphoid infiltrates and more aggressive cutaneous T-cell lymphomas [1,4]. Ancillary studies, including immunohistochemistry and T-cell receptor (TCR) gene rearrangement testing, may support the diagnosis but are not independently diagnostic, as immunophenotypic findings may overlap with other lymphoid proliferations, and TCR clonality does not, by itself, establish malignancy [2,5]. Therefore, diagnosis requires integration of clinical presentation, histopathology, immunophenotype, molecular findings, and clinical course.

We present a case of PCSM-TCLPD with TCR clonality presenting as a solitary nodule on the left forehead, highlighting the importance of clinicopathologic correlation to avoid misdiagnosis and guide appropriate management.

## Case presentation

A 59-year-old Hispanic female presented with a six-month history of a slowly enlarging nodule on the left forehead. The lesion was intermittently pruritic and mildly tender, without bleeding or ulceration. She denied systemic symptoms, including fevers, night sweats, and unintentional weight loss. The patient also reported a similar facial lesion several years earlier that had been surgically excised, though details regarding the diagnosis were unavailable.

**Figure 1:**
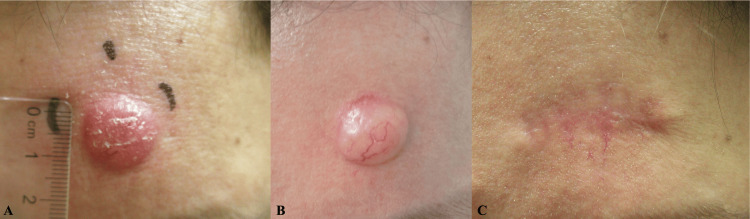
Clinical progression of the lesion. (A) Erythematous, dome-shaped nodule on the left forehead at the time of shave biopsy (~1.5-2.0 cm). (B) Four weeks after shave biopsy, showing regrowth to near the original size. (C) Two months after plastic surgical excision, demonstrating a well-healed scar without evidence of recurrence.

On examination, a solitary erythematous, telangiectatic nodule measuring approximately 1.5 × 2.0 cm was noted on the left forehead (Figure 1A). No other similar cutaneous lesions were noted on examination. The initial clinical differential diagnosis included primary cutaneous B-cell lymphoma, cutaneous metastasis, basal cell carcinoma, and squamous cell carcinoma. A shave biopsy was performed with partial removal of the lesion; however, the nodule regrew to near its original size within four weeks (Figure 1B).

**Figure 2:**
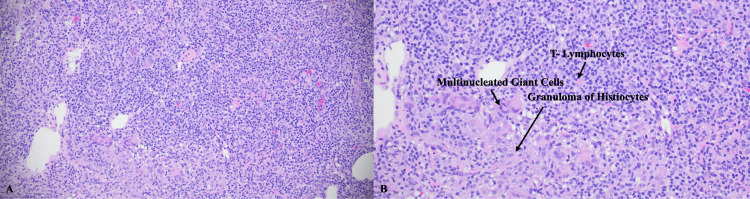
(A) H&E, 20×, showing a dense dermal infiltrate of small/medium lymphocytes (purple-staining cells). (B) Higher magnification highlighting the lymphocytic infiltrate with scattered multinucleated giant cells. H&E: hematoxylin and eosin, MNGC: multinucleated giant cell

**Figure 3:**
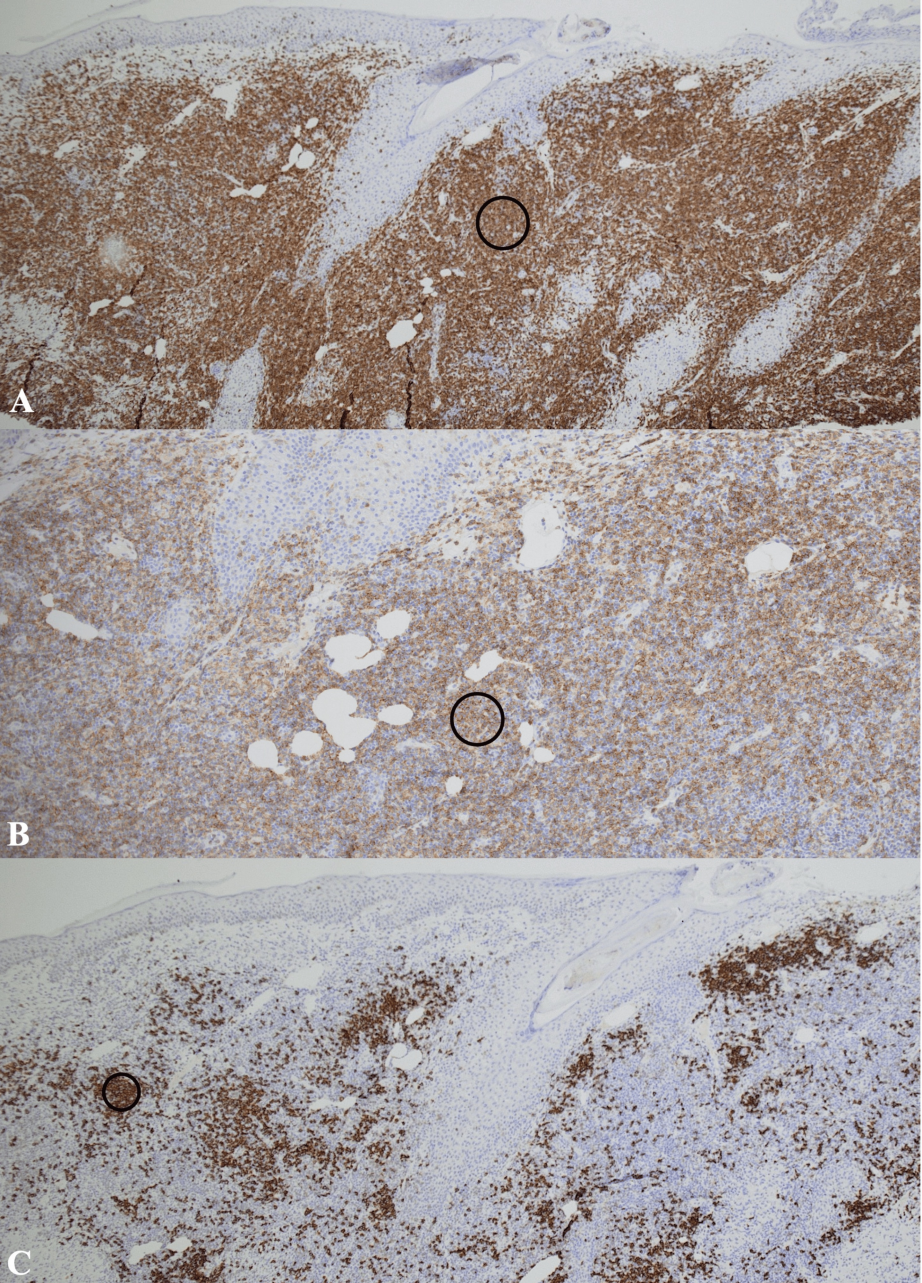
Immunohistochemical profile of the lesion. (A) CD3 stain showing diffuse T-cell positivity throughout the dermal infiltrate. (B) CD4 stain demonstrating predominance of CD4+ T-cells. (C) CD20 stain highlighting scattered B-cell clusters, consistent with admixed reactive B-cells. Circled areas indicate representative regions of positive staining (brown chromogen).

**Figure 4:**
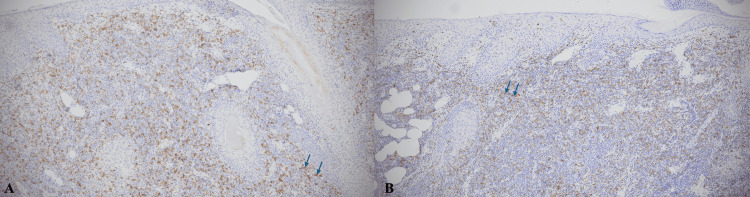
PD-1 (A) and ICOS (B) immunostains showing scattered positive cells (blue arrows) within the dermal infiltrate, with brown chromogen indicating positivity, consistent with a subset of TFH-like elements. PD-1: programmed cell death protein 1, ICOS: inducible T-cell costimulator, TFH: T-follicular helper

Histopathologic examination of the initial biopsy showed a dense dermal infiltrate composed predominantly of small/medium lymphocytes, with scattered histiocytes and multinucleated giant cells, and minimal epidermotropism (Figure 2A-2B). Immunohistochemical analysis (Table 1) demonstrated a predominance of CD3⁺ and CD4⁺ T-cells, with scattered CD20⁺ B-cells, consistent with admixed reactive B-cells (Figure 3A-3C). PD-1 and ICOS expression were present in a subset of cells, supporting a T-follicular helper (TFH) phenotype (Figure 4A-4B). Additional markers are summarized in Table 2, including a low proliferation index (Ki-67 < 20%), favoring an indolent process.

**Table 1:** T-cell marker expression in this case. The immunophenotype (CD2⁺, CD3⁺, CD4⁺, CD8– with rare TFH markers) is characteristic of PCSM-TCLPD, consistent with published reports [1-3]. Staining intensity is reported semi-quantitatively as +++ (diffuse/strong), ++ (moderate), + (focal/weak), or rare (scattered cells). CD: cluster of differentiation, PCSM-TCLPD: primary cutaneous CD4⁺ small/medium T-cell lymphoproliferative disorder, PD-1: programmed cell death protein 1, ICOS: inducible T-cell costimulator, TFH: T-follicular helper

Marker	Expression	Interpretation
CD2, CD3, CD5, CD7	+++	Pan-T-cell markers; loss may suggest atypical, malignant T-cell clones
CD4	+++	Helper T-cell marker; predominates in PCSM-TCLPD
CD8	+	Cytotoxic T-cell marker; CD8+ infiltrates suggest cytotoxic lymphomas or reactive processes
PD-1	++	TFH-associated markers
ICOS	++	TFH-associated markers
CXCL13	Rare	Chemokine marker of TFH cells; supports TFH-derived lymphomas

**Table 2:** Additional marker expression in this case. Findings support a low-proliferation, CD4⁺ T-cell-predominant lymphoproliferative disorder with admixed reactive B-cells rather than a primary B-cell lymphoma or a high-grade process [1-3]. Staining intensity is reported semi-quantitatively as +++ (diffuse/strong), ++ (moderate), + (focal/weak), or rare (scattered cells). CD: cluster of differentiation, BCL6: B-cell lymphoma 6

Marker	Expression	Interpretation
CD10	Rare	Few B germinal center-type cells
CD20	+	Scattered reactive B-cells
BCL6	+	Background B germinal center cells
Ki-67	10-15 % (low <20%)	Low proliferation index supports indolence

Molecular studies demonstrated TCR gamma and beta gene rearrangements, consistent with a clonal T-cell population. Given the historical classification of this entity and the presence of T-cell clonality, the patient was referred to hematology/oncology for staging evaluation. Laboratory studies were largely unremarkable, with no cytopenias or leukocytosis. Mild abnormalities included hyperglycemia, hyperglobulinemia, elevated alkaline phosphatase, and mild hyponatremia, none of which were suggestive of systemic lymphoproliferative disease. CT imaging of the chest, abdomen, pelvis, and neck showed no lymphadenopathy or evidence of systemic disease. Bone marrow biopsy and flow cytometry demonstrated left-shifted granulopoiesis without evidence of hematolymphoid malignancy or plasma cell neoplasm, supporting a localized cutaneous process (Tables 3-5).

**Table 3:** Complete blood count with differential. Complete blood count demonstrating values within normal limits, without cytopenias or leukocytosis, supporting the absence of clinically evident systemic hematologic involvement. Mild hypochromia is reflected by decreased MCH and MCHC. MCH: mean corpuscular hemoglobin, MCHC: mean corpuscular hemoglobin concentration, WBC: white blood cell count, RBC: red blood cell count, HGB: hemoglobin, HCT: hematocrit, MCV: mean corpuscular volume, MCH: mean corpuscular hemoglobin, MCHC: mean corpuscular hemoglobin concentration, PLT: platelet count, MPV: mean platelet volume, RDW: red cell distribution width, Neu %: neutrophil percentage, Neu #: absolute neutrophil count, ANC: absolute neutrophil count, IG %: immature granulocyte percentage, IG #: absolute immature granulocyte count, LY %: lymphocyte percentage, LY #: absolute lymphocyte count, MO %: monocyte percentage, MO #: absolute monocyte count, EO %: eosinophil percentage, EO #: absolute eosinophil count, BA %: basophil percentage, BA #: absolute basophil count, NRBC: nucleated red blood cells, L: low

Test	Result	Units	Flag	Ref range
WBC	7.3	10^3/uL	-	4.8 -10.8
RBC	5.04	10^6/uL	-	4.20-5.40
HGB	13.4	g/dL	-	12.0-16.0
HCT	41.8	%	-	37.0-47.0
MCV	82.9	fL	-	81.0-99.0
MCH	26.6	pg	L	27.0-31.0
MCHC	32.1	g/dL	L	33.0-37.0
PLT	203	10^3/uL	-	130-400
MPV	11.5	fL	-	9.4-12.3
RDW	14.2	%	-	10.5-14.5
Neu %	68.6	%	-	40.0-77.0
Neu # (ANC)	5.02	10^3/uL	-	1.50-6.50
IG %	0.1	%	-	0.0-0.5
IG #	0.01	10^3/uL	-	0.00-0.03
LY %	23.1	%	-	15.0-41.0
LY #	1.69	10^3/uL	-	1.20-3.40
MO %	5.7	%	-	3.0-11.0
MO #	0.42	10^3/uL	-	0.00-1.00
EO %	1.8	%	-	0.0-3.0
EO #	0.13	10^3/uL	-	0.00-0.30
BA %	0.7	%	-	0.0-1.0
BA #	0.05	10^3/uL	-	0.00-0.20
NRBC, %	0	%	-	0.0-0.2
NRBC, absolute, x 10^3/uL	0	10^3/uL	-	0.00-0.01

**Table 4:** Complete metabolic panel. Comprehensive metabolic panel demonstrating mild abnormalities, including hyperglycemia, hyperglobulinemia, elevated alkaline phosphatase, and mild hyponatremia. These findings were nonspecific and were not suggestive of systemic lymphoproliferative disease. L: low, H: high, CO2: carbon dioxide, BUN: blood urea nitrogen, GFR: glomerular filtration rate, A/G ratio: albumin-to-globulin ratio, AST: aspartate aminotransferase, SGOT: serum glutamic-oxaloacetic transaminase, ALT: alanine aminotransferase, SGPT: serum glutamic-pyruvic transaminase

Test	Result	Unit	Flag	Ref range
Sodium	134	mmol/L	L	136-145
Potassium	4.4	mmol/L	-	3.5-5.1
Chloride	98	mmol/L	-	97-107
CO2	28.3	mmol/L	-	21.0-32.0
Glucose	286	mg/dL	H	74-106
BUN	18	mg/dL	-	7-18
Creatinine, mg/dL	1.05	mg/dL	-	0.55-1.30
GFR estimate	61	mil/min/1.73m2	-	>60
BUN/Creatinine ratio	17.1		-	6.0-25.0
Calcium	8.9	mg/dL	-	8.5-10.1
Albumin	3.5	g/dL	-	3.4-5.0
Total protein	7.9	g/dL	-	6.4-8.2
Globulin	4.4	g/dL	H	2.2-4.2
A/G ratio	0.8	-	-	0.8-2.0
Bilirubin, total	0.4	mg/dL	-	0.2-1.0
Alkaline phosphatase	183	U/L	H	46-116
AST/SGOT	23	U/L	-	15-37
ALT/SGPT	31	U/L	-	14-59

**Table 5:** Bone marrow biopsy and flow cytometry findings. Bone marrow flow cytometric analysis demonstrating predominantly mature hematopoietic populations, polyclonal B-cells, a normal CD4:CD8 T-cell ratio, and polyclonal plasma cells. No abnormal or clonal lymphoid or myeloid population is identified, supporting the interpretation of a localized cutaneous process without evidence of systemic hematolymphoid malignancy.

Specimen type	Bone marrow		
Myeloblasts	1.60%		
Hematogones	4.30%		
Polyclonal B-cells	3.20%	Kappa/Lambda ratio = 1.4:1	
Small T-cells	7.80%	CD4:CD8 ratio = 1.5:1	
Natural killer cells	1.20%		
Mature monocytes	3.80%		
Immature monocytes	1.60%		
Neutrophils	68.60%		
Eosinophils	3.40%		
Basophils	0.30%		
Mast cells	0.20%		
Polyclonal plasma cells	0.70%	CyKappa/CyLambda ratio = 1.1:1	
Sample info	Viability	90.00%	
Sample info	Cellularity	35.7 x 10^6 cells/mL	Total specimen volume: 1.5 mL
Markers analyzed	CD2, CD3, CD4, CD5, CD7, CD8, CD10, CD11b, CD13, CD14, CD16, CD19, CD20, CD33, CD34, CD38, CD45, CD56, CD64, CD117, CD123, CD138, HLA-DR, Kappa, Lambda, CyKappa, CyLambda		
Morphology	The morphologic features have been correlated with the flow cytometric findings.		

The patient subsequently underwent definitive excision with plastic surgery. The site healed well, with no evidence of recurrence at the two-month follow-up (Figure 1C). The interval from initial biopsy to definitive excision and follow-up was approximately seven months. Continued clinical follow-up at three- to six-month intervals is planned. A chronological summary of clinical events is provided in Table 6.

**Table 6:** Chronological timeline of clinical events. Clinical timeline summarizing presentation, diagnostic evaluation, systemic workup, treatment, and follow-up in this case of PCSM-TCLPD. PCSM-TCLPD: primary cutaneous CD4+ small/medium T-cell lymphoproliferative disorder, CT: computed tomography

Timepoint	Event
6 months prior to presentation	Slowly growing erythematous forehead nodule
Initial presentation (1st month)	Dermatologic evaluation and shave biopsy performed
1 month later (2nd month)	Pathology results supported PCSM-TCLPD; hematology/oncology referral placed
3rd month	Systemic workup, including laboratory studies, CT imaging, and bone marrow analysis, was negative for systemic disease
5th month	Definitive excision performed by plastic surgery
7th month (2 months post-op)	Follow-up demonstrated a well-healed scar without evidence of recurrence
Ongoing	Clinical follow-up planned every 3-6 months

## Discussion

PCSM-TCLPD is a low-grade cutaneous T-cell lymphoproliferative disorder recognized in the 2018 WHO-EORTC classification [1]. It typically presents as a solitary papule, plaque, or nodule on the head and neck, most often in middle-aged to older adults [1,2]. Multiple clinicopathologic series have described cohorts ranging from approximately 40 to more than 130 patients, along with smaller series and individual case reports [6-8]. The clinical course is generally indolent, with an excellent prognosis and reported five-year survival rates exceeding 95% [1,2].

A key diagnostic challenge is distinguishing PCSM-TCLPD from reactive pseudolymphomas and more aggressive cutaneous T-cell lymphomas [1,2]. Although no formal diagnostic criteria exist, diagnosis relies on integration of clinical morphology, histopathology, immunophenotype, molecular findings, and clinical course [1,2]. TCR gene rearrangement studies may demonstrate clonality; however, clonality is not specific for malignancy and may also be seen in reactive conditions. Therefore, TCR results should be interpreted in the appropriate clinical and histopathologic context [3,6].

In this case, the diagnostic data were interpreted in a structured sequence. Histologically, the lesion showed a dense dermal lymphoid infiltrate composed predominantly of small/medium lymphocytes, with scattered histiocytes and multinucleated giant cells, and minimal epidermotropism (Figure 2). These findings supported a cutaneous lymphoid proliferation but were not independently diagnostic.

Immunohistochemically, the infiltrate showed diffuse expression of pan-T-cell markers, including CD2, CD3, CD5, and CD7, with a strong predominance of CD4 (Figures 3-4; Tables 1-2). This pattern indicated a CD4⁺ T-cell-predominant infiltrate that was consistent with the expected phenotype of PCSM-TCLPD [1,2]. Scattered CD8⁺ cells were also present and were interpreted as a reactive cytotoxic background rather than the dominant lesional population. Expression of TFH-associated markers, including PD-1 and ICOS, with rare CXCL13 positivity, further supported a TFH phenotype previously described in PCSM-TCLPD [1,2,6-8].

Additional immunohistochemical findings helped exclude important mimickers. Scattered CD20⁺ and BCL6⁺ cells were consistent with admixed reactive B-cells rather than a primary B-cell lymphoproliferative process [1,2]. The low Ki-67 proliferation index (<20%) favored an indolent process and argued against a high-grade lymphoid malignancy [1,2]. Molecularly, TCR gamma and beta gene rearrangement studies demonstrated a clonal T-cell population. While this finding supported clonal T-cell proliferation, it was not interpreted in isolation, as T-cell clonality could occasionally be detected in reactive lymphoid infiltrates and, by itself, did not establish malignancy [3,6].

Systemic evaluation was also important in this case. Laboratory evaluation, CT imaging, bone marrow biopsy, and flow cytometry showed no evidence of systemic involvement, lymphadenopathy, cytopenias, leukocytosis, or hematolymphoid malignancy (Tables 2-5). These findings supported a localized cutaneous process rather than systemic lymphoma. Taken together, the clinical presentation of a solitary forehead nodule, a CD4⁺ T-cell-predominant infiltrate with partial TFH marker expression, a low proliferative index, clonal TCR rearrangement, absence of systemic disease, and a benign clinical course supported a diagnosis of PCSM-TCLPD.

In general, management of PCSM-TCLPD is typically conservative, with local therapies such as surgical excision or, less commonly, localized radiotherapy [1,2]. Intralesional corticosteroids and other anti-inflammatory approaches, including doxycycline, have also shown variable success in selected cases [2,4,5,9]. Observation may be appropriate in select patients. Because standardized diagnostic and treatment criteria remain limited, management is often individualized based on clinical presentation, histopathologic findings, clinician judgment, and patient preference [1,2]. Recurrence can occur, particularly after incomplete removal, but systemic dissemination is rare [1,2,7,8], and spontaneous regression has also been reported [2].

## Conclusions

PCSM-TCLPD is an indolent lymphoproliferative disorder that can closely mimic more aggressive cutaneous T-cell lymphomas, making accurate diagnosis essential. Historically classified within the spectrum of cutaneous T-cell lymphoma, this entity has since been redefined to reflect its benign clinical behavior. Recognition of its characteristic clinicopathologic and immunophenotypic features is critical, particularly given that findings such as TCR clonality alone do not establish malignancy.

An integrated clinicopathologic approach is necessary to guide appropriate management and avoid unnecessary interventions. Awareness of its indolent course supports a more conservative, individualized treatment strategy, particularly in cosmetically sensitive areas where preserving function and appearance is an important consideration.
